# Supplementary material for: Paternal country of origin and adverse neonatal outcomes in births to foreign-born women in Norway: A population-based cohort study
Source: PLoS Med. 2020 Nov 4;17(11):e1003395. doi: 10.1371/journal.pmed.1003395 (PMC7641355; doi:10.1371/journal.pmed.1003395)
Supplement: S1 Table — (DOCX) [file pmed.1003395.s002.docx]

| **S1 Table. Associations between paternal identity and adverse neonatal outcomes in births to migrant and Norwegian-born women in Norway (1990-2016).** | | | | | |
| --- | --- | --- | --- | --- | --- |
|  | **Very preterm  (22^+0^-31^+6^ gwks*)**† | **Moderately preterm  (32^+0^-36^+6^ gwks*)**ǂ | **Small for gestational age (SGA)§** | **Apgar score  <7 at 5 minutes**$ | **Stillbirth** |
|  | **Births to migrant women** | | | | |
| **Paternal identity¤** |  | | | | |
| Known father (n) | 222,354 | 230,796 | 233,042 | 237,901 | 238,671 |
| No cases (%) | 2405 (1.1) | 10,847 (4.7) | 28,353 (12.2) | 4164 (1.8) | 1066 (0.4) |
| Reference | 1.00 | 1.00 | 1.00 | 1.00 | 1.00 |
| Unknown father (n) | 1817 | 1769 | 1867 | 1945 | 2088 |
| No cases (%) | 169 (9.3) | 121 (6.8) | 369 (19.7) | 129 (6.6) | 152 (7.3) |
| OR, 95% CI | 9.38 (7.95-11.06) | 1.49 (1.24-1.79) | 1.78 (1.58-2.00) | 3.99 (3.33-4.78) | 17.50 (14.68-20.86) |
| P-value | <0.001 | <0.001 | <0.001 | <0.001 | <0.001 |
| aOR, 95% CI¶ | 10.36 (8.04-13.36) | 1.26 (1.03-1.54) | 1.35 (1.18-1.54) | 3.26 (2.61-4.06) | 16.62 (13.62-20.28) |
| P-value | <0.001 | 0.023 | <0.001 | <0.001 | <0.001 |
|  | **Births to Norwegian-born women** | | | | |
| **Paternal identity¤** |  | | | | |
| Known father (n) | 1,128,662 | 1,169,994 | 1,180,077 | 1,220,000 | 1,226,168 |
| No cases (%) | 10,909 (1.0) | 52,241 (4.5) | 96,838 (8.2) | 16,304 (1.3) | 4712 (0.4) |
| Reference | 1.00 | 1.00 | 1.00 | 1.00 | 1.00 |
| Unknown father (n) | 5108 | 4842 | 5287 | 5468 | 6159 |
| No cases (%) | 716 (14.0) | 450 (9.3) | 966 (18.2) | 439 (8.0) | 664 (10.8) |
| OR, 95% CI | 16.70 (15.39-18.13) | 2.19 (1.99-2.42) | 2.50 (2.33-2.68) | 6.44 (5.84-7.12) | 31.32 (28.74-34.14) |
| P-value | <0.001 | <0.001 | <0.001 | <0.001 | <0.001 |
| aOR, 95% CI¶ | 15.43 (13.63-17.46) | 1.58 (1.42-1.75) | 1.49 (1.38-1.60) | 5.77 (5.09-6.55) | 28.52 (25.83-31.49) |
| P-value | <0.001 | <0.001 | <0.001 | <0.001 | <0.001 |
| * Gestational weeks  † Cases with missing data on gestational age (migrant, n=5620; Norwegian-born, n=45,866) and moderately preterm births (migrant, n=10,968; Norwegian-born, n=52,961) excluded  ǂ Cases with missing data on gestational age (migrant, n=5620; n=45,866) and very preterm births (migrant, n=2574; Norwegian-born, n=11,625) excluded  § Cases with missing data on SGA excluded (migrant, n=5850; Norwegian-born, n=46,963)  $ Cases with missing data on Apgar score excluded (migrant, n=913; Norwegian-born, n=6859)  ¶ Adjusted for year of birth, parity, maternal age, paternal age, marital status, maternal education and mother’s gross income. Adding marital status to the regression models for stillbirth reduced goodness of fit, and the variable was therefore omitted from these analyses.  ¤ A known father may be either foreign-born or Norwegian-born | | | | | |
