# Supplementary material for: Paternal country of origin and adverse neonatal outcomes in births to foreign-born women in Norway: A population-based cohort study
Source: PLoS Med. 2020 Nov 4;17(11):e1003395. doi: 10.1371/journal.pmed.1003395 (PMC7641355; doi:10.1371/journal.pmed.1003395)
Supplement: Attachment — Submitted filename: Response to reviewers.docx [file pmed.1003395.s003.docx]

Bergen 02 July 2020

**Response to reviewers**

We are grateful for the valuable comments and suggestions on the manuscript entitled “Associations between paternal origin and adverse neonatal outcomes in births to migrant women: a Norwegian population-based study" (PMEDICINE-D-20-01868R1) for consideration at PLOS Medicine, which have helped us to improve our paper. Please find our responses and comments below. We hope you find the revised manuscript suitable for publication.

**NOTES FROM THE AUTHORS:**

In this letter, our response to reviewers are written in blue. Changes in the manuscript are written in red.

**Reviewer #1:**

**Note to reviewer #1:**

Please note that in response to comments from reviewer #2, we no longer stratify the results on primiparous and multiparous women. In addition, as requested by both reviewers #2 and #3, we have now also analyzed data for Norwegian-born (non-migrant women), provided in a separate table.

First, the authors should not use causal language (e.g. line 49 "influence" but other places too). This is an observational study, causation cannot be inferred.

**Response:** We agree with the reviewer and have now rephrased any causal language that can be potentially misleading for readers.

Second, the dependent variable for gestation should be gestation time in days or weeks and a spline can be used to evaluate nonlinearity. But, if only the categorized data is available, then ordinal logistic regression should be used. Similarly, Apgar score should not be categorized. Doing so makes an Apgar of 1 the same as one of 6 and a score of 7 the same as 10. That makes no sense.

**Response:** We understand the reviewer’s concerns and have now reanalyzed gestational age and Apgar scores as continuous outcomes using linear regression models. The association measures are reported in the table below (Table X) as crude and adjusted difference in means (with 95% CI and P values) between comparison groups (i.e. the Norwegian-born, foreign-born, unregistered). Although group difference in means may be informative for some readers, our initial categorization of Apgar (Committee on Obstetric Practice American Academy of Pediatrics—Committee on Fetus and Newborn, 2015; Ehrenstein, 2009) and preterm birth (Blencowe et al., 2013; Blencowe et al., 2012) is very common in the medical literature, more easily interpreted and clinically relevant. We, therefore, prefer to keep the categorized analyses as reported in the original manuscript tables and figures. In addition, note that the analysis (and approximate likelihood ratio test) of preterm birth using ordinal logistic regression suggests that odds ratios were not proportional across categories (which also can be seen from the original results for preterm categories). We have therefore also kept results from the separate binary logistic regression analysis for preterm categories in the original tables.

| **Table X. Associations between paternal origin as well as identity, and Apgar Score at five minutes. Analyzed by linear regression.** | | | | | | |
| --- | --- | --- | --- | --- | --- | --- |
|  | **Unadjusted regression** |  | **Adjusted regression*** |  | **Adjusted regression*** |  |
|  | **B (95% CI)** | **P value** | **B (95% CI)** | **P value** | **B (95% CI)** | **P value** |
| **Paternal origin** | | | | | | |
| Norwegian-born | Reference |  |  |  |  |  |
| Foreign-born | 0.004 (-0.006; 0.013) | 0.442 | -0.200 (-0.298; -0.102) | <0.0001 |  |  |
| Unregistered | -0.119 (-0.142; 0.097) | <0.0001 | -0.167 (-0.191; -0.144) | <0.0001 |  |  |
| **Paternal identity** | | | | | | |
| Known | Reference |  |  |  |  |  |
| Unknown identity | -0.439 (-0.534; -0.344) | <0.0001 |  |  | -0.399 (-0.498; -0.300) | <0.0001 |
|  |  |  |  |  |  |  |
| * Adjusted for year of birth, parity, maternal age, paternal age, maternal education, mother’s gross income and marital status | | | | | | |

Third, independent variables should also not be categorized. In *Regression Modelling Strategies* Frank Harrell lists 11 problems with this and sums up "nothing could be more disastrous". Leave age and length of residence in years.

**Response:** Thank you for pointing this out. Our original models included two covariates that can be replaced by their original continuous scales, namely year of birth and maternal age. Further, in response to comments from reviewer #3, we have now also included paternal age as a continuous term in the regression models. To account for non-linear relations with the outcomes, the three covariates were included as quadratic polynomial terms (e.g., age + age^2). To avoid collinearity between first and second order terms, all covariates were centered at their means before entering into the models. The reanalysis using continuous covariates (including paternal age) did not alter results and conclusions. The analysis description and tables have been updated (lines: 194-195).

Fourth, for maternal and paternal origin, the reference group should be Norwegian. First, this is both the largest group and the "norm". Second, using foreign born as the reference puts the other two levels below and above the reference. It is better to have the lowest (or highest) category as the reference.

**Response:** We agree with the reviewer. We have now provided the data using Norwegian born fathers as the reference category, which has enhanced the readability and ease of interpretation. Tables and text have been updated accordingly.

**Reviewer #2:**

General comments

The association between missing information and adverse health outcomes (and pregnancy outcomes in particular) has been described previously in many studies. The authors should include a wider description and discussion of the unknown paternal identity and unregistered paternal origin to ensure that readers understand exactly what the researchers studied. The difference between unknown paternal identity, unregistered paternal origin and single mother need to be discussed. That is one of the main focus of the paper, but the authors should clarify the social, health and clinical aspects behind those variables: single mother? not access to social and health rights? …. The unreported father is a proxy for more noteworthy factors.

**Response:** Thank you for this valuable comment. We have added one Australian reference to the introduction (line: 111). In the Methods, we have added information that we hope will help the reader to understand the difference between the two exposure variables (paternal origin and paternal identity) (line: 156-167). Likewise, similar information is now provided as footnotes to relevant tables (Table 1 and S1 Table). Further, we have changed our approach related to the discussion on paternal identity as this variable, obtained from the national birth registry, has not been investigated previously in relation to adverse neonatal outcomes in migrant women. We have also added marital status as an adjustment variable in the main analyses. We hope these changes have added value to the study.

Some methodological details should be expanded and clarified.

-	I was wondering if the missing data were MNAR (Missing not at random) . Did you check if there were associated to adverse outcome? We know that missing data for sociodemographic data are more important among stillbirths. If the data are MNAR, the multiple imputation is more complex, and some assumptions need to be verified (check with a statistician)

**Response:** We believe the missing data mechanism for education and income is likely to be MAR. The main reason for this is that the level of education and income largely depend on what country the women are born in, and to some extent also to the age of the women and the year of giving birth. It should also be noted that, except for a few census surveys performed in Norway during the study period, educational data from Statistics Norway are only available if a woman has educated herself in Norway and was registered by the Norwegian educational system. Although this may explain the higher missing proportion for immigrant women, by using administrative data there is no apparent systematic selection in that women with a particular educational level could refrain from providing information on their education level. Given this and that education is strongly related to maternal country of birth, we believe that the missing data mechanism for educational level in our data may be largely MAR, and to a lesser extent MNAR. We would argue similarly for the income data.

-	For the logistic regression, did you check the Goodness of fit of the models and interaction tests?

**Response:** We have now performed Hosmer and Lemeshow’s goodness-of-fit tests for all analyses involving immigrant women. The tests were performed on adjusted regression models by using one of the 5 complete data sets obtained by multiple imputations. The test was done for 5, 10, and 15 quantile groups, respectively. All p values were high across tests for all analyses, except for the analyses of paternal identify and stillbirth. For this analysis, we performed the Lemeshow’s goodness-of-fit tests for various models with different subsets of explanatory variables, and discovered that model fit was satisfactory only after omitting marital status. Consequently, we did not include marital status as an adjustment variable in the analyses of paternal identity and stillbirth. Accordingly, a note on this has been added as a footnote to S1 Table.

-	The stratification primiparous/multiparous need to be discussed because the profiles are similar. The interaction test could justify the choice of stratification rather than adjustment for parity.

**Response:** The strategy of analyzing data separately for primiparous and multiparous women was chosen a priori before analyses. Our initial argument was that Norwegian primiparous and multiparous women are often provided with different recommendations in antenatal guidelines and we therefore believed that this difference could affect associations differently for these groups. However, we agree with the reviewer that there is no strong evidence that the associations differ between these groups, particularly for the main analyses of paternal origin. Accordingly, we have now reanalyzed the associations using all data with no stratification on primiparous and multiparous women. We have updated the text and tables accordingly. Associations did not differ importantly across primiparous and multiparous women (lines: 205-211).

-	Maternal factors as single mother and maternal length of residence are mentioned in the methodology but not analyzed? Why ?

**Response:** Thank you for spotting this. Initially, the two variables were included for description purposes only, but were then left out during the final manuscript preparation to reduce the number of words.

We did not adjust for any of these variables. In our opinion, length of residence is an intermediate variable, and should not be treated as an adjustment variable for the association between paternal origin and health outcomes in the present study. However, if the variable were to be adjusted for, it would have little impact on the association of paternal origin or identity with the various neonatal outcomes (please see table below, Table XX). In light of this, we prefer to leave length of residence out from the analyses.

We initially made no adjustment for single status because we believed a priori that this variable could be collinear with paternal identity, and possibly also with paternal origin. Although this seemed obvious at the time of analysis, we now see that the variable does not cause major problems for most analyses, except for the analysis of paternal identity and stillbirth, indicated by poor goodness-of-fit test and the large jump in effect estimate (see table below). Consequently, we adjusted for marital status in all analyses except for the analyses of paternal identity and stillbirth.

| **Table XX. Associations between paternal factors (paternal origin and paternal identity) and adverse neonatal outcomes in births to migrant women in Norway (1990-2016).** | | | | |
| --- | --- | --- | --- | --- |
|  | **Very preterm  (22+0-31+6 gwks*)**† | **Moderately preterm  (32+0-36+6 gwks*)**ǂ | **Apgar score  <7 at 5 minutes**§ | **Stillbirth** |
| **Paternal origin** |  |  |  |  |
| Norwegian-born (n) | 64,818 | 67,369 | 69,246 | 69,477 |
| no cases (%) | 615 (1.0) | 3166 (4.7) | 1043 (1.5) | 229 (0.3) |
| Reference | 1.00 | 1.00 | 1.00 | 1.00 |
| Foreign-born (n) | 141,697 | 146,911 | 151,538 | 152,097 |
| no cases (%) | 1573 (1.1) | 6787 (4.6) | 2606 (1.7) | 695 (0.5) |
| OR, 95% CI | 1.17 (1.06-1.29) | 0.98 (0.94-1.03) | 1.14 (1.06-1.23) | 1.39 (1.19-1.61) |
| aOR, 95% CI\| | 1.24 (1.12-1.38) | 1.00 (0.95-1.05) | 1.17 (1.08-1.26) | 1.37 (1.17-1.61) |
| aOR, 95% CI¶ | 1.20 (1.08-1.33) | 0.98 (0.93-1.03) | 1.14 (1.05-1.23) | 1.26 (1.08-1.48) |
| Unregistered (n) | 17,656 | 18,285 | 19,062 | 19,185 |
| no cases (%) | 386 (2.2) | 1015 (5.6) | 644 (3.4) | 294 (1.5) |
| OR, 95% CI | 2.33 (2.05-2.66) | 1.19 (1.11-1.28) | 2.29 (2.07-2.53) | 4.71 (3.96-5.59) |
| aOR, 95% CI\| | 2.49 (2.15-2.87) | 1.19 (1.10-1.29) | 2.31 (2.07-2.58) | 4.86 (3.99-5.91) |
| aOR, 95% CI¶ | 2.29 (1.97-2.66) | 1.15 (1.06-1.25) | 2.23 (1.99-1.50) | 4.87 (3.98-5.96) |
| **Paternal identity** |  |  |  |  |
| Known father (n) | 222,354 | 230,796 | 237,901 | 238,671 |
| no cases (%) | 2405 (1.8) | 10,847 (4.7) | 4164 (1.8) | 1066 (0.5) |
| Reference | 1.00 | 1.00 | 1.00 | 1.00 |
| Unknown father (n) | 1817 | 1769 | 1945 | 2088 |
| no cases (%) | 169 (9.3) | 121 (6.8) | 129 (6.8) | 152 (7.3) |
| OR, 95% CI | 9.38 (7.95-11.06) | 1.49 (1.24-1.79) | 3.99 (3.33-4.78) | 17.50 (14.68-20.86) |
| aOR, 95% CI\| | 8.81 (7.39-10.49) | 1.32 (1.10-1.60) | 3.51 (2.90-4.24) | 16.55 (13.56-20.20) |
| aOR, 95% CI¶ | 10.36 (8.04-13.36) | 1.26 (1.03-1.54) | 3.26 (2-61-4.06) | 31.42 (23.35-42.29) |
| * Gestational weeks  † Cases with missing data on gestational age (n=5620) and moderately preterm births (n=10,968) excluded  ǂ Cases with missing data on gestational age (n=5620) and very preterm births (n=2574) excluded  § Cases with missing data on Apgar score excluded (n=913)  \| Adjusted for year of birth, parity, maternal age, paternal age, maternal education, mother’s gross income and length of residence  ¶ Adjusted for \|and marital status, except length of residence | | | | |

Weakness of the discussion

The authors should include more information in the discussion part to clarify some points to avoid confusion.

-	This study requires a comparison with the non-migrant population. The unknown paternal identity and unregistered paternal origin is probably also associated to increased OR for adverse outcome among non-migrant population. The results do not fully support a conclusion specific to migrant population.

**Response:** We agree with the reviewer. In a separate analysis, we have now analyzed data also for Norwegian born non-migrant women. Foreign paternal origin was not associated with adverse neonatal outcomes in births to non-migrant women. However, the association between unregistered paternal origin and neonatal outcomes applied to both migrant and non-migrant women. This, of course, led to some changes in the text description of the study sample, statistics, results, and interpretation. Thank you for your advice.

-	P16 line 267: the discussion for sub-Saharan Africa women can be generalized for other migrant group (literacy, distrust healthcare system, delay antenatal care,..) because it is not specific to sub-saharan Africa mother. Other studies have shown the high proportion of single mother among sub-Saharan African mother.

**Response:** Thank you for this valuable comment. The text has been updated accordingly (lines: 378-382).

**Reviewer #3:**

This manuscript covers the important issue of paternal origin and migrant primip and multip mothers in conjunction with prematurity, low Apgar score, and stillbirth in Norway. The results when controlling for maternal factors of age, education, and income are not surprising, mothers with foreign-born fathers had a higher risk of very preterm and stillbirth compared with Norwegian-born fathers (regardless of multip or primip mothers). Paternal foreign-born status was also associated with lower Apgar score compared to Norwegian-born fathers. Additionally, unknown paternal origin was linked to higher risk of all adverse birth outcomes for both primip and multip mothers. Finally, also expected, unknown father identity was associated with adverse birth outcomes compared with identified father, regardless of primip or multip mother.

The dearth of research on the impact of fathers on birth outcomes makes this a very important contribution to the literature. Furthermore, given the disparity in prenatal care given as noted in the Methods section, hopefully this further highlights the existing inequity and empowers policy makers and providers to move toward solutions in Norway and beyond.

There are several major issues that do need to be addressed including why the models did not adjust for any paternal factors, why small for gestational age was not included as an outcome, and why the authors excluded the Norwegian born mothers rather than using them in a comparison with migrant mothers.

Major issues (although the authors may not be able to do all the analyses mentioned, it would be helpful to 1. Add analyses, 2. Add to a limitation, or 3. Note this is a future direction of the research team):

1. The authors note they do not have access to paternal education which does have an independent effect on birth outcomes. Yet they have paternal age and do not adjust for this factor also known to be associated with birth outcomes, especially stillbirth.

**Response:** Thank you for this comment. In the updated version of the manuscript, analyses are adjusted for paternal age.

2. Further unlike advanced maternal age which is typically defined as 35 years, advanced paternal age is typically defined at 40. It may be beneficial to add this as a category and further examine this in conjunction with migrant mothers and paternal foreign-born status. Also why no assessment of teen aged parents either mother or father (less than 20?), as these lower extremes of ages have also been associated with poor birth outcomes.

**Response:** Thank you for this advice. We have now included both paternal and maternal age as adjustment variables. To avoid loss of statistical power and information, the variables were included as quadratic polynomial terms and not as categorical variables.

3. It is unclear why small for gestational age (a birth outcome previously having been associated with fathers and stress) was not assessed. Could this be added?

**Response:** Thank you for this comment. We acknowledge that differences in birthweight may reflect maternal and infant health problems or suboptimal care. However, infants may be growth restricted for a variety of reasons and in relation to migration and the risk of adverse neonatal outcomes, the difference in birthweight may just as well be attributed to normal biological variation. Further, data available in the Medical Birth Registry of Norway do not include specific information on growth restriction, merely infant birthweight. The interpretation of differences in birthweight as pathological or as merely reflecting normal variability is not straightforward (Urquia, Sørbye, & Wanigaratne, 2016), thus we chose not to pursue associations between paternal origin and small for gestational age in the current paper.

4. Another interesting outcome that should be considered if available is NICU admission. NICU admission is more impactful than low apgar score which is not clearly associated with nor predictive of poor neonatal outcomes.

**Response:** Thank you for this comment. Initially, we did consider investigating associations between paternal origin and NICU admission. However, in Norway, there are large differences in local practices related to NICU admission and therefore we chose not to pursue associations between paternal origin and NICU admission in the current paper.

5. It was also unclear why the authors excluded the Norwegian born mothers. Couldn't this group be assessed for paternal foreign born or not and the outcomes could be instructive in relation to migrant mothers and paternal foreign born status. Additionally, outcomes for the general population as a benchmark would also be important to know for generalizability.

**Response:** Thank you for this advice. This was also suggested by reviewer 2. We have now also analyzed the data for Norwegian born non-immigrant women. The associations of unregistered paternal origin and unknown paternal identity with neonatal outcomes were also found for the non-immigrant women. We have updated the text description of the study sample, statistics, results, and interpretation accordingly.

6. Additionally, why was both income and education included in the models, often these two variables are co-linear? Was this assessed? Are they both necessary in the models?

**Response:** It is well-known that many migrants do not achieve levels of income/employment in their new country that are commensurate with their high education levels. The Spearman’s correlation coefficient between education and income in the dataset of immigrants was 0.34, suggesting there is no collinearity. For this reason, we think it is wise to keep both variables in the analyses, although they had little impact on the association measures.

7. There is no discussion of race and ethnicity. In the US, there is the "healthy immigrant" effect, possible as certain races/ethnicities have not had the weathering from endemic racism. Could a migrant mother and foreign born father have additional discrimination from their race/ethnicity aside from country of origin? Was there data on this? Also consider referencing Collins work (one reference is Collins JW Jr, Wu SY, David RJ. Differing intergenerational birth weights among the descendants of US-born and foreign-born Whites and African Americans in Illinois. Am J Epidemiol. 2002; 155:210-6.)

**Response:** Thank you for this comment. We had no access to information on race or ethnicity. Without such data we felt it would be unwise to speculate about race or ethnicity in the current paper. We have now discussed some of the findings in relation to the healthy migrant effect in the discussion (lines: 347-350).

8. How long were the mothers and father in Norway for and why was this not adjusted for as the authors mention they calculate maternal length of residence and categorize it, but I did not see that data nor why this was not in the models.

**Response:** Thank you, this was also spotted by reviewer 2. Initially, the length of residence was included for descriptive purposes only, but was then left out during the final manuscript preparation to reduce the number of words. In our opinion, this variable is an intermediate variable and should not be treated as an adjustment variable for the association between paternal origin and health outcomes in present study. However, if adjustments were to be made, it would have little impact on the association of paternal origin or identity with the various neonatal outcomes (please see table in response to reviewer 2).

Other Minor Edits/By Section:

Abstract

1. Results - Line 46-48 "Unregistered paternal origin and unknown paternal identity were both associated with increased odds of adverse neonatal outcomes." Please clarify in comparison with?

**Response:** Thank you for this comment. The manuscript has been changed in line with this suggestion (lines 61-65).

Introduction

1. The introduction seems to simplify a very complex body of literature where paternal identification and origin is intertwined with marital status, race/ethnicity, social economic status and support. This study approaches this by separating out countries of origin of the mother. However, there should be some acknowledgement of additional known paternal effects and birth outcomes.

**Response:** Thank you for this comment. We have added new aspects and references to the introduction in relation to this comment (lines: 108-113).

2. I believe the introduction would be strengthened by more explicitly delineating the gaps in literature, how this study will address the gaps in a novel way in the Introduction.

**Response:** Thank you for this comment. The manuscript has been changed accordingly (lines 114-116).

Methods:

1. Could the choices of what did and did not go into the models be more detailed? Why not consanguinity or paternal age?

**Response:** Thank you for this comment. We have added literature regarding paternal age to the introduction part of the manuscript, and analyses are now adjusted for paternal age (lines: 111-112). We chose not to include consanguinity to the statistical models because consanguinity was rare and so were the adverse outcomes. Adjusting for consanguinity would therefore not have altered the results notably.

2. Can you explain the exclusion factor of second-generation migrant women, does this mean the women were excluded if they were born in Norway but their parents were not or does it mean that the migrant woman was foreign born but her parents were from Norway? What does women with mixed background mean? One parent was from Norway and one not?

**Response:** Thank you for this comment. We have addressed this issue in the manuscript under *Study population* (lines: 140-143).

3. The information provided in the Setting Section of the Methods was very helpful.

**Response:** Thank you.

4. It might be helpful to provide examples of which high income countries are most common locations for migrants to originate from.

**Response:** We have added information on the most common countries represented in the study (lines: 220-231).

5. Is there data on previous preterm birth-a big predictor of preterm delivery in the future among the multi-parous, if not should be listed in Discussion as limitation.

**Response:** We have added a sentence about this under limitations (lines: 312-313).

6. Any data on maternal high risk pregnancy? Pre-existing morbidities OR pregnancy related diseases-preclampsia or diabetes or obesity that could increase risk of stillbirth and rate of prematurity? If not, should be a limitation in Discussion

**Response:** Thank you for this comment. We did have access to some of the factors mentioned. However, these data were either not systematically collected for the whole period (i. e. maternal obesity), were considered mediating factors (i.e. pregnancy complications) or were considered low prevalent conditions that would be less likely to affect the point estimates (i.e. prepregnancy diabetes).

7. What about substance use, especially tobacco use which may be higher or lower in certain countries mothers and fathers migrate from? If not, should be in Discussion.

**Response:** We have added a sentence about this under limitations (lines: 312-318).

8. Why not report in addition to very and moderately preterm rates, the overall preterm rate?

**Response:** Thank you for this comment. We chose to report preterm rates by categories because the clinical value of reporting an overall preterm rate is limited.

Results:

1. No bivariate analyses of frequencies reported, only OR and aOR. However, would be helpful to know if the frequencies differ among groups. Were the data in Table1, Figure 2 compared/analyzed? If so should be denoted

**Response:** Thank you for this comment. Tables and text have been updated accordingly.

2. Why do authors think slightly increased risk of Norwegian-born fathers before adjustment of having moderate preterm birth?

**Response:** Thank you for this comment. We have added a short paragraph discussing this particular finding (lines: 332-338).

3. For Table 3 Does known father mean known and could be either foreign born or norwegian? If so these might be separated? Either way should be clarified.

**Response:** Thank you for this comment. A known father may be either foreign-born or Norwegian-born. A footnote explaining this is added to relevant tables (Table 1 and S1 Table).

Discussion:

1. I think the Discussion could benefit to some restructuring such that limitations are noted together in serial paragraphs where limitations are not just listed but as to why this study is still valid despite the limitations. Foloowed by a strengths paragraph explaining why this study is well designed, novel and important contribution. The current strength paragraph is mixed with limitations. I have noted above some possible limitations to explore.

**Response:** We have added several of your suggestions concerning limitations to the manuscript. We hope these additions have added value to the discussion (lines: 312-318).

2. Line 264 which notes that the US study only examined twins. There are other US studies not only of twins. One example is Ma S. Paternal Race/Ethnicity and Birth Outcomes. December 2008, Vol 98, No. 12 | American Journal of Public Health.

**Response:** Thank you for this comment. The study mentioned is interesting; however, our main focus is on migration, not race or ethnicity. We did however add an Australian study to the introduction and discussion parts of the manuscript (line: 110).

3. It may be helpful to discuss more of the biological underpinings of the findings-toxic stress and related inflamation which can lead to poor birth outcomes, how support from a father regardless of marriage may mitigate the inflammation etc.

**Response:** Thank you for this comment. It would have been interesting to discuss more of the biological underpinnings of the findings, however, biological aspects are mentioned, and we do not have data supporting further in-depth discussion of such mechanisms.

4. Do the authors surmise that a father from the host population makes a difference due to anything specific to infrastructure in Norway that might not exist in the countries that published the studies the authors cite as background? If so this might also help the reader know why this study is different/may be novel?

**Response:** Thank you for this comment. We believe that possible explanations for a better outcome associated with a Norwegian-born father compared to a foreign-born father in births to migrant women are appropriately discussed (lines: 342-347). However, we have included Norwegian-born women in separate analyses. Further, we also added information on the ten most common paternal countries of births represented in the study, for both migrant and Norwegian-born women (lines: 220-231). We have added a few comments related to these changes in the manuscript, in the discussion part of the paper (lines: 347-350, 361-364, 366-370). To avoid adding speculation not supported by the findings or other literature, we think it is appropriate not to add more to this part of the discussion. We hope you find this acceptable.

**Yours sincerely,**

Eline Skirnisdottir Vik, RN RM PhD-candidate (eline.skirnisdottir.vik@hvl.no)

Blencowe, H., Cousens, S., Chou, D., Oestergaard, M., Say, L., Moller, A.-B., . . . Group, t. B. T. S. P. B. A. (2013). Born Too Soon: The global epidemiology of 15 million preterm births. *Reproductive Health, 10*(1), S2. doi:<https://doi.org/10.1186/1742-4755-10-S1-S2>

Blencowe, H., Cousens, S., Oestergaard, M. Z., Chou, D., Moller, A.-B., Narwal, R., . . . Say, L. (2012). National, regional, and worldwide estimates of preterm birth rates in the year 2010 with time trends since 1990 for selected countries: a systematic analysis and implications. *The Lancet, 379*(9832), 2162-2172. doi:<https://doi.org/10.1016/S0140-6736(12)60820-4>

Committee on Obstetric Practice American Academy of Pediatrics—Committee on Fetus and Newborn. (2015). The Apgar score (Committee Opinion No. 644). *Obstetrics & Gynecology, 126*(4), 52-55. doi:<https://doi.org/10.1097/AOG.0000000000001108>

Ehrenstein, V. (2009). Association of Apgar scores with death and neurologic disability. *Clinical epidemiology, 1*, 45. doi:<https://doi.org/10.2147/CLEP.S4782>

Urquia, M. L., Sørbye, I. K., & Wanigaratne, S. (2016). Birth-weight charts and immigrant populations: A critical review. *Best Practice & Research Clinical Obstetrics & Gynaecology, 32*, 69-76. doi:<https://doi.org/10.1016/j.bpobgyn.2015.09.001>
