# Supplementary material for: Paternal country of origin and adverse neonatal outcomes in births to foreign-born women in Norway: A population-based cohort study
Source: PLoS Med. 2020 Nov 4;17(11):e1003395. doi: 10.1371/journal.pmed.1003395 (PMC7641355; doi:10.1371/journal.pmed.1003395)
Supplement: Attachment — Submitted filename: Response to reviewer.docx [file pmed.1003395.s004.docx]

Bergen 12 August 2020

**Response to reviewer**

We are grateful for the valuable comments and suggestions on the manuscript entitled “Associations between paternal origin and adverse neonatal outcomes in births to migrant women: a Norwegian population-based study" (PMEDICINE-D-20-01868R1) for consideration at PLOS Medicine, which have helped us to improve our paper. Please find our responses and comments below. We hope you find the revised manuscript suitable for publication.

**NOTES FROM THE AUTHORS:**

In this letter, our response to reviewer is written in blue. Changes in the manuscript are written in red.

**Reviewer #3:**

The authors have done a significant amount of work to respond well to my critiques, especially adding the Norwegian mothers. I still have one major concern remaining:

1.	I strongly urge the authors to reconsider their lack of analysis of size for gestational age. Similar to their argument for the concerns of birthweight for gestational age, prematurity is multi-factorial (maternal social factors, maternal genetic factors, maternal chronic disease/overall health, fetal health). Regardless, in general, it is accepted that small for gestational age is a poor birth outcome as prematurity is, and both have been linked to not only maternal factors but also paternal factors. In fact, in the Introduction section, the authors refer to low birth weight (less informative that size for gestational age) as an adverse birth outcome. There are very few birth outcomes based on vital statistics to assess, and size for gestational age or even term low birth weight are both meaningful. The authors have an opportunity to study birthweight for gestational age in this unique population. In fact, just recently, these same authors using a migrant

mother/Norwegian birth cohort and birth outcomes similar to this manuscript (but without paternal information) also examined SGA (small for gestational age) status (Vik et al. Country of first birth and neonatal outcomes in migrant and Norwegian-born parous women in Norway: a population based study. BMC Health Services Research (2020) 20:540). It would be interesting for the authors to refer to their BMC Health Services Research publication and compare/contrast SGA and other birth outcome in the current Results and Discussion sections as well.

**Response:** As requested by the reviewer, we have now included the analyses for small for gestational age (SGA). The manuscript text (Abstract, Author summary, Methods, Results, Discussion and Conclusion) and Tables has been updated accordingly.

**Yours sincerely,**

Eline Skirnisdottir Vik, RN RM PhD-candidate (eline.skirnisdottir.vik@hvl.no)
